# The role of the transient receptor potential ankyrin type-1 (TRPA1) channel in migraine pain: evaluation in an animal model

**DOI:** 10.1186/s10194-017-0804-4

**Published:** 2017-09-07

**Authors:** Chiara Demartini, Cristina Tassorelli, Anna Maria Zanaboni, Germana Tonsi, Oscar Francesconi, Cristina Nativi, Rosaria Greco

**Affiliations:** 1 Laboratory of Neurophysiology of Integrative Autonomic Systems, Headache Science Center, “C. Mondino” National Neurological Institute, Pavia, Italy; 2 Department of Brain and Behavioral Sciences University of Pavia, Pavia, Italy; 3 Department of Chemistry ‘Ugo Schiff’, University of Florence, Florence, Italy; 4 FiorGen, University of Florence, Florence, Italy; 5 IRCCS “National Neurological Institute C. Mondino” Foundation, Via Mondino, 2, 27100 Pavia, Italy

**Keywords:** NTG, Migraine, Trigeminal hyperalgesia, TRPA1 antagonist

## Abstract

**Background:**

Clinical and experimental studies have pointed to the possible involvement of the transient receptor potential ankyrin type-1 (TRPA1) channels in migraine pain. In this study, we aimed to further investigate the role of these channels in an animal model of migraine using a novel TRPA1 antagonist, ADM_12, as a probe.

**Methods:**

The effects of ADM_12 on nitroglycerin-induced hyperalgesia at the trigeminal level were investigated in male rats using the quantification of nocifensive behavior in the orofacial formalin test. The expression levels of the genes coding for c-Fos, TRPA1, calcitonin gene-related peptide (CGRP) and substance P (SP) in peripheral and central areas relevant for migraine pain were analyzed. CGRP and SP protein immunoreactivity was also evaluated in trigeminal nucleus caudalis (TNC).

**Results:**

In rats bearing nitroglycerin-induced hyperalgesia, ADM_12 showed an anti-hyperalgesic effect in the second phase of the orofacial formalin test. This effect was associated to a significant inhibition of nitroglycerin-induced increase in c-Fos, TRPA1 and neuropeptides mRNA levels in medulla-pons area, in the cervical spinal cord and in the trigeminal ganglion. No differences between groups were seen as regards CGRP and SP protein expression in the TNC.

**Conclusions:**

These findings support a critical involvement of TRPA1 channels in the pathophysiology of migraine, and show their active role in counteracting hyperalgesia at the trigeminal level.

## Background

Migraine is a neurovascular disease characterized by recurrent attacks of predominantly unilateral throbbing head pain. Trigeminovascular system activation, followed by dural neurogenic inflammation and sensitization phenomenon, seems to be one of the main mechanisms that underlie migraine attacks. Preclinical and clinical data support a role for several mediators, such as calcitonin gene-related peptide (CGRP) in migraine pathophysiology, and highlight the pharmacological agents that target these mediators for migraine treatment. Transient receptor potential (TRP) channels are a large family of non-selective cation channels that are important in pain signaling pathways. Several findings show that TRPs are important in migraine pain and associated symptoms, including hyperalgesia and allodynia [1]. The thermo-TRP ankyrin type-1 (TRPA1) channels - sensors of oxidative, nitrative and electrophilic stress – have been involved in different models of pain diseases [2] and seem to play a key role also in the mechanisms of migraine pain. Indeed, their activation is operated by a plethora of exogenous and endogenous stimuli, among which there are many migraine triggers [1, 3, 4]. TRPA1 channels can be found in many types of cells and tissue, but they are mostly expressed in sensory neurons [5, 6]; they are implicated in meningeal nociceptive and vascular responses involving neurogenic dural vasodilatation and plasma extravasation. TRPA1 are co-expressed with TRP vanilloid type-1 (TRPV1) channels in nociceptive neurons, where they trigger or enhance neurotransmitter release [5, 7, 8]. In analogy to TRPV1 channels, TRPA1 channels can detect pungent plant compounds, are modulated by temperature and components of the inflammatory environment and are upregulated during pain and inflammation [9]. Indeed, it seems likely that these TRP channels show functional and physical interactions [10].

In the trigeminovascular system, TRPA1 channels activation induces CGRP release from trigeminal neurons and dural tissue and stimulate meningeal vasodilatation [8, 11–14]. In addition, TRPA1 agonists cause the activation of second order neurons in the trigeminal nucleus caudalis (TNC) [15], suggesting their role in the initiation of migraine attacks. Thus, the involvement of TRPA1 is emerging as a major contributing pathway in migraine, though studies on the importance of this TRP channel in the pathophysiology of migraine, and in particular in the trigeminal hyperalgesia, are still limited.

In this study, we evaluated the role of TRPA1 channel in trigeminal hyperalgesia in a well validated animal model of migraine, based on nitroglycerin (NTG) administration [16–19] in association to the orofacial formalin test [20, 21]. More specifically, we evaluated the effect of a TRPA1 antagonist, AMD_12, on the behavioral, neurochemical and transcriptional components of the model.

## Methods

### Animals

In this study we used adult male Sprague-Dawley rats (weight 200-250 g) following the IASP’s guidelines for pain research in animals [22]. Rats were housed in plastic boxes in groups of 2 with water and food available ad libitum and kept on a 12:12 h light-dark cycle. All procedures were in accordance with the European Convention for Care and Use of Laboratory Animals and were approved by the Italian Ministry of Health (Document number 1239/2015PR).

### Drugs

Nitroglycerin (NTG) (Bioindustria L.I.M. Novi Ligure (AL), Italy) was prepared from a stock solution of 5.0 mg/1.5 mL dissolved in 27% alcohol and 73% propylene glycol. For the injections, NTG was further diluted in saline (0.9% NaCl) to reach the final concentration of alcohol 6% and propylene glycol 16%. The diluted NTG is injected intraperitoneally (i.p.) at the dose of 10 mg/Kg, [16, 20, 21]. An equivalent volume of saline (0.9% NaCl), alcohol 6% and propylene glycol 16% was used as vehicle. The TRPA1 antagonist ADM_12, synthesized in the Laboratory of Prof. Cristina Nativi (University of Florence, Italy) and characterized by a high binding constant versus TRPA1 [23], was dissolved in saline and administered i.p. at the dose of 30 mg/Kg.

### Experimental plan

The animals were allocated in four groups, formed by 13 animals each, and randomly assigned to different experimental set according to the experimental protocol illustrated in Table 1. Briefly, ADM_12/saline was administered 3 h after NTG/vehicle treatment; 4 h after NTG/vehicle administration [16], the rats underwent the orofacial formalin test [20, 21]. All animals were acclimatized to the test chamber 30 min before testing. At the end of the behavioral test, each rat was sacrificed with a lethal dose of anesthetic (Chloral hydrate 800 mg/kg, i.p., and Tiletamine-Zolazepam, 50 mg/Kg, intramuscular) and their brain and cervical spinal cord were removed and processed either for the detection of expression levels of the genes encoding for c-Fos (c-fos), TRPA1 (Trpa1), CGRP (Calca) and Substance P (SP) (preprotachykinin-A, PPT-A) with real time polymerase chain reaction (RT-PCR) or for the quantification of CGRP and SP expression with immunohistochemistry (IHC).

**Table 1:** Schematic representation of experimental groups, timing (T) of administration and number (N) of animals *per* group assigned to different experimental set

Experimental group	T0	T3 h	T4 h	RT-PCR	IHC
Control (CT)	NTG vehicle	saline	formalin	*N* = 7	*N* = 6
ADM	NTG vehicle	ADM_12	formalin	*N* = 7	*N* = 6
NTG	NTG	saline	formalin	*N* = 7	*N* = 6
NTG + ADM	NTG	ADM_12	formalin	*N* = 7	*N* = 6

*RT-PCR* real time PCR, *IHC* immunohistochemistry

An a priori power analysis was conducted to determine the minimal sample size needed to obtain a statistical power of 0.80 at an alpha level of 0.05. In our previous study [20] we evaluated the difference of at least 20% in nociceptive response in the second phase of the orofacial formalin test (time of face rubbing) between rats injected with NTG and rats injected with vehicle (NTG vehicle) and we calculated a standardized effect size of 1.683 for this variable. The power analysis by GPower 3.1 estimated a sample size of at least 6 rats for experimental group.

### Orofacial formalin test

The subcutaneous injection of formalin (1.5%, 50 μl), an aqueous solution of 37% formaldehyde, was performed into the right upper lip, with minimal animal restraints. Immediately after the injection, each animal (*N* = 13 per group) was placed into the observation box (30x30x30-cm glass chamber with mirrored sides) and rubbing behavior was recorded for 45 min with a camera, located at 50 cm from the box, for the off-line analysis. Pain-related behavior, linked to the trigeminal activation, was quantified by measuring the seconds the animal spent grooming the injected area (face rubbing) with the ipsilateral fore- or hindpaw. The observation time was divided into 15 blocks of 3 min each for the time course analysis [24]. The test consisted of 2 phases spaced by a latency period of 6–12 min: Phase I (0–6 min) refers to the acute pain, while Phase II (12–45 min) reflects the combined effects of afferent input and central sensitization [24]. Analysis of the rubbing behavior was made by an investigator who was blinded to the animal’s group assignment.

After completion of the orofacial formalin test, a subset of 7 rats per experimental group served for the evaluation of gene expression by means of RT- PCR, while the remaining subset of 6 animals per experimental group underwent evaluation of protein expression by means of immunohistochemistry.

### RT- PCR

The trigeminal ganglion (TG) and cervical spinal cord (CSC) ipsilateral to the formalin injection and medulla-pons in toto of each animal were removed and processed to evaluate expression levels of the genes encoding for c-Fos (c-fos), TRPA1 (Trpa1), CGRP (Calca) and SP (PPT-A). mRNA expression was analyzed by a RT-PCR as previously described [21, 25, 26]. Total RNA was extracted from samples with TRIzol® (Invitrogen, USA), in combination with tissue homogenization by means of ceramic beads (PRECELLYS, Berthin Pharma). RNA quality was assessed using a nanodrop spectrophotometer (Euroclone); cDNA was generated using the iScript cDNA Synthesis kit (BIO-RAD) following the supplier’s instructions. Gene expression was analyzed using the Fast Eva Green supermix (BIO-RAD). Primer sequences, obtained from the AutoPrime software (http://www.autoprime.de/AutoPrimeWeb), are reported in Table 2. The expression of the housekeeping gene, glyceraldehyde 3-phosphate dehydrogenase (GAPDH), remained constant in all the experimental groups considered. The amplification was performed through two-step cycling (95–60 °C) for 45 cycles with a light Cycler 480 Instrument RT-PCR Detection System (Roche) following the supplier’s instructions. All samples were assayed in triplicate and the ΔΔCq method was used to investigate the differences in the gene expression levels.

**Table 2:** Sequences of primers used

Gene	Forward primer	Reverse primer
GAPDH	AACCTGCCAAGTATGATGAC	GGAGTTGCTGTTGAAGTCA
c-fos	TACGCTCCAAGCGGAGAC	TTTCCTTCTCTTTCAGTAGATTGG
Trpa1	CTCCCCGAGTGCATGAAAGT	TGCATATACGCGGGGATGTC
Calca	CAGTCTCAGCTCCAAGTCATC	TTCCAAGGTTGACCTCAAAG
PPT-A	GCTCTTTATGGGCATGGTC	GGGTTTATTTACGCCTTCTTTC

### Immunohistochemical staining

After the behavioral test the animals, belonging to a second experimental set, were anaesthetized and perfused transcardially with saline and 4% paraformaldehyde. The medullary segment containing the TNC between +1 and −5 mm from the obex was removed, post-fixed for 24 h in the same fixative and subsequently transferred in solutions of sucrose at increasing concentrations (up to 30%) during the following 72 h. All samples were cut transversely at 30 μm on a freezing sliding microtome. CGRP and SP protein expression was evaluated in the TNC ipsilaterally to the formalin injection using the free-floating immunohistochemical technique. Following several rinses in a potassium phosphate buffered saline (KPBS) solution, sections were incubated in a blocking solution (4% normal goat serum) for 30 min; subsequently, sections were incubated in primary antibodies in a KPBS solution containing 0.4% Triton X-100 (TX) and 4% normal goat/horse serum for 24 h at room temperature. For CGRP we used an anti-rabbit antibody (Santa Cruz Biotechnology, Santa Cruz, CA, USA) at a dilution of 1:3200. For SP we used an anti-rabbit antibody (Chemicon, Temecula, CA, USA) at a dilution of 1:5000. After several rinses in a KPBS solution containing 0.04% TX, sections were incubated at room temperature with the secondary biotinylated antibody (Vector Laboratories, Burlingame, CA, USA) and then with the avidin–biotin complex (Vectastain, Vector Laboratories). Peroxidase substrate kit DAB (3′3′-diaminobenzidine tetrahydrochloride) (Vector Laboratories, Burlingame, CA, USA) was used for visualization.

Negative control staining was performed by omitting the primary antibodies and, in order to avoid variability in the background staining due to the procedure, each treated animal was simultaneously stained with the corresponding control and processed at the same time. After staining, sections were rinsed in KPBS, mounted onto glass slides, air dried and cover slipped.

### Statistical evaluation

Statistical analysis was performed with GraphPad Prism program (GraphPad Software, San Diego, CA). For the orofacial formalin test, the time spent (in seconds) in face rubbing was counted separately for Phase I and for Phase II. For mRNA expression, results were analyzed using the ΔCt method to compare expression of genes of interest with that of GAPDH. The area covered by CGRP and SP immunoreactive fibres in the TNC ipsilateral to the formalin injection, was expressed as optical density (OD) values, as suggested by previous reports [21, 27]. OD was obtained using an AxioSkop 2 microscope (Zeiss) and a computerized image analysis system (AxioCam, Zeiss), equipped with dedicated software (AxioVision Rel 4.2, Zeiss, Germany). The mean OD was determined by rounding off the stained structure of interest (TNC) and subtracting the OD of the background (slide, mounting medium and coverslip) for each section, considering a total of 12 sections per animal. All sections were averaged and reported as the mean ± SEM of OD values.

All data were tested for normality using the Kolmogorov-Smirnov (K-S) normality test and considered normal. Differences between groups were analyzed by the one-way analysis of variance (ANOVA) followed by Tukey’s Multiple Comparison Test. A probability level of less than 5% was regarded as significant.

## Results

### ADM_12 effect on behavioral response

**Fig. 1:**
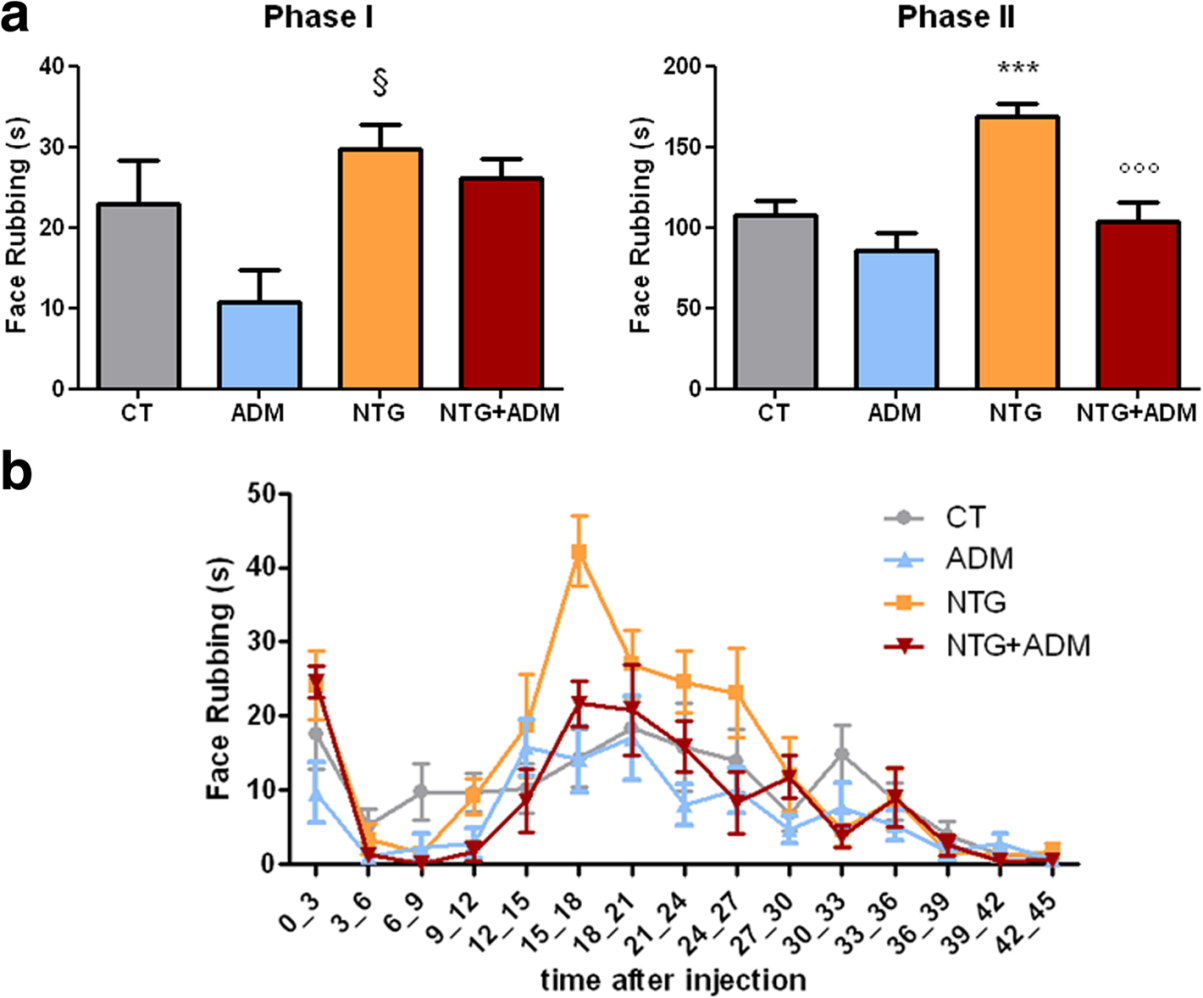
Orofacial formalin test. **a** Total time (seconds) spent in face rubbing in Phases I and II. NTG administration significantly increased nocifensive behavior in Phase II as compared to control group (CT). No difference was found in Phase I. ADM_12 administration did not provoke any significant changes during either phase of the test when used with NTG vehicle (ADM group). When ADM_12 was administered in association with NTG (NTG + ADM group), it prevented NTG-induced increase in nocifensive behavior in Phase II. **b** Time course of the face rubbing. Data are expressed as mean ± SEM. ANOVA followed by Tukey’s Multiple Comparison Test, §*p* < 0.05 vs ADM; ***p < 0.001 vs CT and ADM; °°°*p* < 0.001 vs NTG

In agreement with our previous findings [20, 21], NTG administration significantly increased nocifensive behavior in Phase II (hyperalgesic phase) of the orofacial formalin test, when compared to control group (CT) (Fig. 1a). No difference was found in Phase I. ADM_12 administration induced a not significant reduction of the nocifensive behavior only during Phase I of test when used alone; on the contrary, when it was administered in association with NTG (NTG + ADM group), it significantly reduced the face rubbing time during Phase II when compared to NTG group (Fig. 1a).

### ADM_12 effect on gene expression

**Fig. 2:**
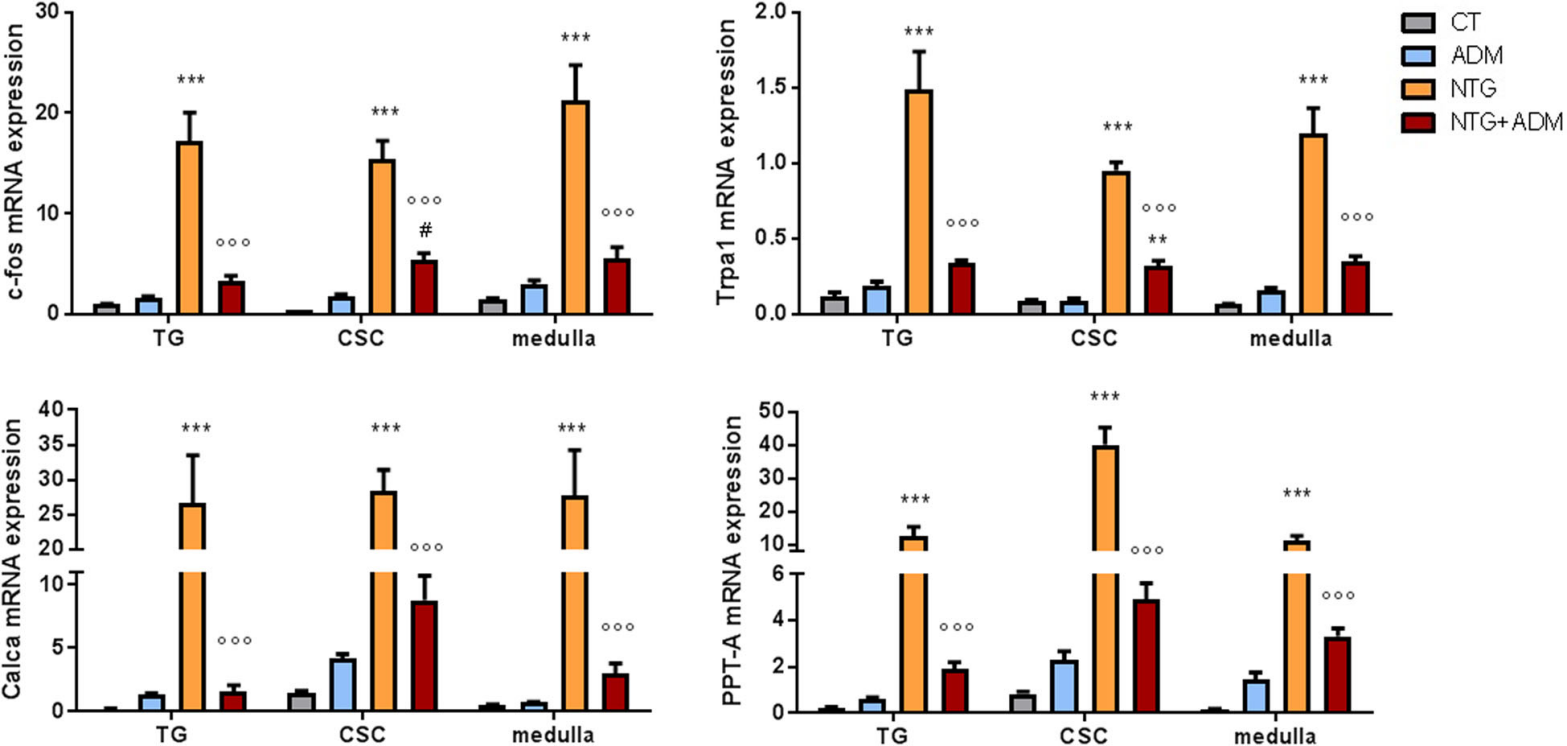
c-fos, Trpa1, Calca and PPT-A mRNA expression. NTG administration caused a significant increase in the expression of all genes in trigeminal ganglion (TG) and cervical spinal cord (CSC) ipsilateral to the formalin injection, and in medulla-pons (medulla) in toto compared to control group (CT). ADM_12 administration (NTG + ADM group) prevented NTG-induced increase of all four genes in all the areas. No change was observed in gene expression levels when ADM_12 was administered in association with NTG vehicle (ADM group). Data are expressed as mean ± SEM. ANOVA followed by Tukey’s Multiple Comparison Test, **p < 0.01 and ***p < 0.001 vs CT and ADM; °°°*p* < 0.001 vs NTG; #*p* < 0.05 vs ADM

In all areas under evaluation, NTG administration caused a significant increase in c-fos and Trpa1 gene expression as well as for the mRNA levels of CGRP (Calca) and SP (PPT-A) (Fig. 2), compared to CT group. ADM_12 treatment prevented the NTG-induced increasing of all genes evaluated in all areas (Fig. 2). No change was observed in gene expression levels when ADM_12 was administered in association with NTG vehicle (Fig. 2).

### ADM_12 effect on CGRP and SP protein expression in TNC

**Fig. 3:**
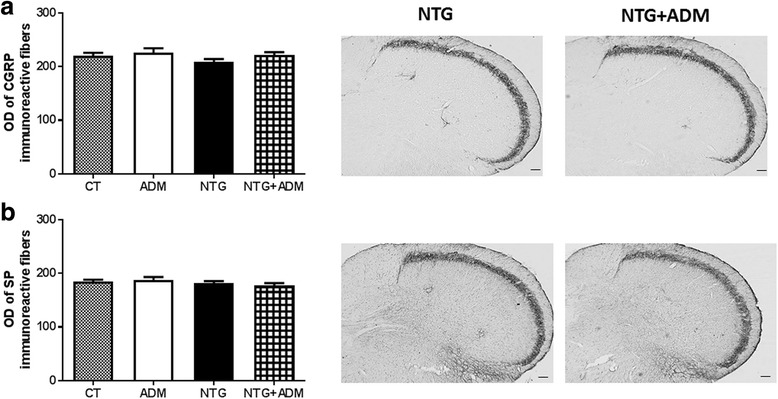
Optical density (OD) values of CGRP and SP immunoreactive fibers in the TNC ipsilateral to the formalin injection. No significant differences were seen between NTG and CT groups for either CGRP (**a**) or SP (**b**). ADM_12 administration did not provoke any change in CGRP (**a**) and SP (**b**) expression either when associated to NTG vehicle (ADM group) or in combination with NTG (NTG + ADM group). Right: representative photomicrographs of CGRP (**a**) and SP (**b**) immunoreactive fibers in NTG and NTG + ADM group. Scale bar: 100 μm

No significant difference in the density of immunoreactive fibers for CGRP and SP protein in the TNC ipsilateral to the formalin injection was observed when comparing NTG and CT groups (Fig. 3). ADM_12 administration did not provoke any change in CGRP and SP expression neither with NTG vehicle nor in combination with NTG (Fig. 3).

## Discussion

Clinical and experimental studies have pointed to the possible involvement of the TRPA1 channels in migraine pain [2, 12]. The activation of TRPA1, expressed on primary sensory neurons, leads to the release of SP and CGRP, key neuropeptides implicated in the trigeminovascular system activation [28]. In this study, we investigated the effects of ADM_12, a TRPA1 antagonist, in NTG-induced hyperalgesia at the trigeminal level in rats [16, 29, 30], using the quantification of nocifensive behavior induced by the orofacial formalin test. Additionally, we analyzed the mRNA expression of the genes coding for c-Fos (c-fos), TRPA1 (Trpa1), CGRP (Calca) and SP (PPT-A) in specific peripheral and central areas involved in trigeminal nociception. CGRP and SP protein expression was also investigated in the TNC.

Our findings show the ability of ADM_12 to reduce NTG-induced hyperalgesia [16, 29, 30] in the second phase of the orofacial formalin test. This effect is associated to a significant inhibition of NTG-induced increase in c-fos, Trpa1, Calca and PPT-A mRNA levels in medulla-pons, ipsilateral cervical spinal cord (CSC) and ipsilateral trigeminal ganglion (TG). By contrast, ADM_12 did not influence gene expression when used in animals that were not made hyperalgesic by NTG treatment, and induced only a moderate, not significant reduction in the nocifensive behavior during Phase I of the orofacial formalin test. Since formalin is able to activate TRPA1 channels [31], this reduction is probably linked to the antagonist action of ADM_12 on the TRPA1 channels localized at the peripheral endings of the primary sensory neurons or on non-neuronal cells.

### C-Fos and TRPA1 mRNA expression

The protein and mRNA c-fos expression is commonly used as a marker of neuronal activation following painful stimuli [17, 20, 32, 33]. Orofacial formalin injection induces an increase in c-Fos mRNA levels in medulla-pons, CSC and TG when compared to orofacial saline injection (data not shown). Here, we show that NTG injection induces a further amplification of primary and second order neurons activation, as demonstrated by c-Fos mRNA levels in TG and in central areas compared to CT group. NTG is able indeed to activate and sensitize spinal trigeminal neurons [18, 20, 21, 34]. Probably, the increase in inflammatory response (e.g. TNF-α, IL-6) [21, 26] induced by NTG in all areas investigated, contributes to the intensification of c-Fos expression [35, 36]. The treatment with the TRPA1 antagonist reverts these changes. Interestingly, the selective TRPA1 antagonist HC-030031 downregulates IL-6 and PGE2 production, confirming that TRPA1 may play a role in the upregulation of these inflammatory factors [37].

TRPA1 mRNA levels are increased in all areas involved in trigeminal nociception after NTG administration. The mechanisms underlying NTG-induced hyperalgesia are believed to depend upon an increased availability of nitric oxide (NO), either released directly from the drug [38] or synthetized ex novo in the meninges [39]. Increased availability of NO would in turn stimulate trigeminovascular terminals to induce inflammation [21, 26, 40], and possibly to upregulate TRPA1 channels [41]. It is known that the cysteine residues of TRPA1 channels are target of NO and NO nitrosylation [42] could contribute to channels sensitization, which in turn would amplify neuropeptides release [43]. On the other hand, it is also known that pro-inflammatory agents activate and/or sensitize nociceptors by means of TRPA1 [44] and their stimulation causes the release of neuropeptides.

Our data confirm the contribution of TRPA1 to NTG-induced hyperalgesia. In agreement, other groups showed that antisense mRNA for TRPA1 prevents carrageenan-induced inflammatory hyperalgesia, suggesting that channel activation is necessary for both the development and the maintenance of hyperalgesia [45]. The understanding of the molecular mechanisms involved in the regulation of these TRP channels expression is limited. However, Hatano et al. [46] have suggested that TRPA1 gene expression is induced via the nuclear factor-κB (NF-κB) signaling. In this frame, therefore, it is possible that ADM_12 administration caused a reduction of calcium (Ca2+) influx through TRPA1 channels, which in turn interfered with the cascade of second-messenger molecules (e.g. via the phospholipase C/Ca^2+^ signaling pathway) and with the Ca^2+^-interacting proteins [47, 48], ultimately preventing NTG-induced NF-kB activation [40].

### Neuropeptides expression

SP and neurokinin A are encoded by the gene PPT-A. Neurokinins exert a variety of biological activities including nociception, synaptic transmission (as excitatory neurotransmitters), and neurogenic inflammation. In particular, SP is the best characterized of these neuropeptides and has been shown to be related to nociceptive (pain) responses and neurogenic inflammation. SP often coexists and is co-released with CGRP [49] and glutamate [50] in the TG and TNC. It is known that in response to prolonged noxious stimuli, SP and CGRP are released from trigeminal sensory nerve fibers around dural blood vessels, leading to endothelium dependent vasodilation, increased microvascular permeability, and plasma and protein extravasation. TRPA1 channels in primary sensory neurons frequently co-localize with CGRP and SP [5, 51] and their activation after NTG administration could promote neuron depolarization and the consequent biosynthesis and releasing of neuropeptides. TRPA1 channels are also expressed on a multitude of non-neuronal sites. Formalin injection may indeed induce TRPA1 activation in various cell types including keratinocytes [52], which may release a large variety of different mediators to indirectly activate and/or sensitize primary sensory neurons [53, 54]. Therefore, it can be hypothesized that a direct effect on TG or at central level may occur as well as an indirect activation via exposed keratinocytes or other cells in the nerve endings proximity. TRPA1 channels have also been described in macrophages [55, 56], thus it is also possible an effect of ADM_12 on these cells by inhibition of NF-κB activation and other inflammatory mediators that may interact with sensory nerves to affect pain and neurogenic inflammation.

Here, we report a significant increasing in Calca and PPT-A mRNA expression in the evaluated areas 4 h after NTG, while no difference in protein expression was found at the same time point in the TNC. This apparently paradoxical finding may be related to compensatory mechanisms aimed at reintegrating CGRP and SP stores after these neuropeptides have been released at the trigeminovascular endings subsequently to the NTG administration [27]. In a previous study, we detected a reduction in CGRP-immunoreactivity (ir) that occurred from the 1st until the 4th hour after NTG administration, while SP-ir increased transiently 1 h after NTG administration and returned to baselined levels at the 4th hour [27]. In the present study, we have evaluated CGRP and SP protein expression 5 h after NTG administration, it is therefore possible that the discrepancy observed is related to the different timings required for the different biological processes (synthesis of mRNA on one side and storage of newly synthetized peptides on the other) and to the different time of evaluation (4 h in our previous study, 5 h in the present one). Other studies show an increase in CGRP release from the TG neurons during neurogenic inflammation or after NO donor treatments together with an increased CGRP gene transcription [57, 58]. In addition, stimulation of peripheral afferent fibers causes SP release within the trigeminal ganglia and this release is greatly amplified following orofacial inflammation [59]. Similarly, a significant increasing in the expression of SP was found 2 h after orofacial formalin test in the TNC [60]. Altogether, our results reinforce the role of SP and CGRP in persistent pain by acting at both peripheral and central levels. In the experimental condition of NTG-induced hyperalgesia, the increased levels of NO and the release of inflammatory agents can sensitize the TRPA1 channels, thus amplifying neuropeptides release. In this frame, the inhibitory effect of ADM_12 on Calca and PPT-A mRNA expression suggests its capability to reduce NTG-induced formation/release of neuropeptides. In line with our observations, Nakamura et al., [61] demonstrated that TRPA1 activation evokes SP release from the primary sensory neurons through phosphorylation of p38 mitogen-activated protein kinase, via an increase in intracellular Ca^2+^, and inflammatory responses.

### Limitations of the study and future perspectives

A time course of the biological responses investigated in the present study would have added important information on the observed changes. However, this type of study would have imposed a marked increase of animal groups. Furthermore, we knew from our previous observations [16, 18] that the maximal intensity of NTG-induced hyperalgesia occurs 4 h after its administration. For this reason, we decided to focus our attention on the 4 h time point.

As regards the ADM_12 treatment, it would be interesting to address further studies with different time points and dosages, and to assess its preventive efficacy on the NTG-induced hyperalgesia as well. Moreover, a study with different time points would give more information about the activity of ADM_12 in baseline condition.

## Conclusions

Taken together our data show that TRPA1 channels play a key role in the behavioral responses associated with pain hypersensitivity induced by NTG at trigeminal level and suggest that SP/neurokinin A and CGRP release may contribute to the central and peripheral sensitization phenomenon. Moreover, the ADM_12 treatment could be a promising tool to counteract hyperalgesia and probably also migraine pain.

## Abbreviations

CGRP: Calcitonin gene-related peptide
CSC: Cervical spinal cord
KPBS: Potassium phosphate buffered saline
NF-κB: Nuclear factor-κB
NO: Nitric oxide
NTG: Nitroglycerin
PPT-A: Preprotachykinin-A
SP: Substance P
TG: Trigeminal ganglion
TNC: Trigeminal nucleus caudalis
TRP: Transient receptor potential
TRPA1: Transient receptor potential ankyrin type-1
TRPV1: Transient receptor potential vanilloid type-1
